# Urgent Colectomies for Cancer: Evaluating the Role of Specialized Colorectal Surgeons

**DOI:** 10.7759/cureus.83030

**Published:** 2025-04-26

**Authors:** Lior Orbach, Shiran Gabay, Tal Montekio, Yehuda Kariv, Meir Zemel, Adam Abu-Abeid, Matan Shimron, Yonatan Lessing, Guy Lahat, Jonathan B Yuval

**Affiliations:** 1 General Surgery, Tel Aviv Sourasky Medical Center, Tel Aviv, ISR; 2 Faculty of Medicine, Tel Aviv University, Tel Aviv, ISR

**Keywords:** acute care surgery and trauma, clinical expertise, colorectal cancer, general and colorectal surgeon, mesenteric lymphadenectomy, surgical oncology, urgent surgeries

## Abstract

Background

Urgent colectomies constitute a significant portion of acute care surgery (ACS). While general surgeons (GS) typically perform colonic resections, more complex cases, particularly those involving colorectal cancer (CRC) and inflammatory bowel disease, may demand a higher level of expertise. This study examines the outcomes of CRC-related urgent colectomies with end stoma performed by colorectal specialist (CRS) surgeons compared to those conducted by GS without sub-specialization or with sub-specialization other than CRS.

Methods

This study analyzed data from patients who underwent emergent colectomy with end stoma due to complications related to colon cancer at a single tertiary hospital between 2013 and 2023. Patients were grouped according to the presence of a CRS surgeon in the surgery. The primary outcomes measured were 90-day perioperative complications, including mortality. Secondary outcomes included quality metrics of the resected specimen as well as oncological outcomes.

Results

The study included 98 patients undergoing emergent colon resection with end stoma, with a mean age of 70.05 ± 14.6 years and a gender distribution of 60 females to 38 males. The mean Charlson comorbidity score was 4.01 ± 2.6. Colorectal surgeons were present in the operations of 32 patients (32.7%). No significant differences were found in baseline characteristics between the groups. The 90-day complication rate was similar between the CRS and GS groups (40.6% vs. 36.9%, p=0.724), as was the rate of major complications (Clavien-Dindo (CD) ≥3, 12.5% vs. 19.7%, p=0.378). The 90-day mortality rate was lower in the CRS group (6.45% vs. 15.1%), but this difference was not statistically significant (p=0.327). The number of lymph nodes (LN) in the specimen was significantly higher in the CRS group (24.6 ± 13.4 vs. 19.1 ± 8.8, p=0.019); however, inadequate lymphadenectomy rate (defined as fewer than 12 LN) did not differ between the groups (16.7% GS vs. 15.6% CRS, p=0.991). Positive margins were rare, with all occurrences found in the GS group (four radial, one distal, 7.6% vs. 0%, p=0.11). Kaplan-Meier survival curves indicated no significant difference in overall survival (log-rank p=0.236) over a median follow-up of 1.5±2.67 years, though the CRS group's curve was consistently higher than that of the GS group.

Conclusion

Urgent colectomies related to colon cancer show comparable outcomes when performed by GS and CRS, with the exception of a higher lymphadenectomy yield in the CRS group. Given the limitations of the study's small sample size and retrospective design, which may result in underpowering, there is a potential trend toward improved survival and reduced major complication rates for the CRS group that did not reach statistical significance. Future research should investigate this issue in a larger, prospective study.

## Introduction

Specialized surgical care has increasingly garnered attention in modern medicine, driven by trends toward field-specific specialization in surgical practice [1,2]. It is widely established that the volume of surgeries is directly linked to patient outcomes on both surgeon- and hospital-specific bases, with high volume correlated with better results due to refined expertise and experience [3-5]. Very high-volume centers exhibit mortality absolute risk reduction of 2% for colonic resections [4]. In particular, colorectal surgery outcomes are shown to be highly dependent on the surgeon’s level of specialization, with colorectal specialist (CRS) surgeons outperforming general surgeons (GS) in complex cases such as those involving colorectal cancer (CRC) and inflammatory bowel disease (IBD) [1,6]. This relationship between surgeon specialization and outcomes has influenced surgical training programs and led to a growing preference for subspecialized care in the management of colorectal diseases, even in emergency settings [7].

Acute care surgery (ACS), which frequently involves high-risk cases such as urgent colonic resections, has long been associated with increased morbidity and mortality compared to elective surgeries. An American College of Surgeons National Surgical Quality Improvement Program (ACS NSQIP)-based study shows an excess 30-day mortality and complication rates of approximately 10% and 21%, respectively [8]. These excess risks are often attributed to the emergent nature of these cases, patient comorbidities, and the need for immediate intervention at suboptimal physiologic status. Acute care surgery is, therefore, an ideal target for quality improvement initiatives [9]. Despite efforts to mitigate these risks, studies have demonstrated that outcomes in ACS are not solely determined by preoperative risk factors but are also significantly influenced by the surgeon’s experience and specialization [10]. The introduction of ACS services over the past decades has aimed to address this issue, optimizing the management of emergency cases by creating a specialized service dedicated to acute settings with a profound impact on improved complication rates, shorter length of stay (LOS), and reduced hospitalization costs [11].

While ACS services have demonstrated improvements in patient outcomes, particularly for common urgent general surgery procedures [12], their role and efficacy are debatable regarding cases requiring more complex, disease-specific management, such as colorectal emergencies due to malignancy and IBD [10,13-15]. Studies have shown that colonic resections performed by acute care surgeons can achieve results equivalent to those performed by colorectal specialists in certain scenarios, particularly for non-oncologic emergencies [16]. However, the scope of ACS training does not fully encompass the oncologic principles and advanced disease-specific management techniques and decision pathways that are central to colorectal surgery. Additionally, long-term oncologic outcome data regarding recurrence and survival are lacking for the appropriate evaluation of a specialization's impact on patient prognosis. 

The aim of this study is to compare the perioperative, specimen quality, and oncological outcomes of emergency colectomies for complicated CRC performed by GS and those performed by CRS. Given that emergency colectomies represent a significant portion of ACS [17], understanding the impact of surgeon specialization on patient outcomes is crucial [15]. We hypothesize that while general surgeons are proficient in performing these procedures, colorectal specialists may achieve better results due to their focused training and higher surgical volumes of colonic resections.

## Materials and methods

Using MDClone software (MDClone Ltd., Beer Sheva, ISR), a digital inquiry was utilized to retrospectively identify and establish a database composed of patients above 18 years of age who underwent emergent colectomy with end stoma due to cancer-related obstruction or perforation between 2013 and 2023, including right colectomy, sigmoid and left colectomy, and subtotal colectomy. Patients with non-CRC-related resections, non-urgent semi-elective settings, improper procedure code classification (e.g., take-back for elective colectomies, diversion ostomy surgeries), and insufficient documentation, as well as pregnancy, were excluded. Patients were grouped according to the specialization level of the attending leading the index operation, i.e., GS vs. CRS. Colorectal specialist surgeons were defined as attending surgeons who had completed a formal colorectal fellowship in North America or Europe. The study protocol was approved by the Tel Aviv Sourasky Medical Center Institutional Review Board with a waiver of informed consent (approval no. TLV-0156-23).

Relevant baseline data were collected from the electronic medical record and included patient age, sex, Charlson comorbidity index, physical examination findings and laboratory results in the emergency department (ED), and indication for surgery. The primary outcome measured was 90-day complications with grade ≥ three according to the Clavien-Dindo (CD) classification [18]. Secondary outcomes included all other complications, quality metrics of the resected specimen, including lymph node yield (LNY), the rate of positive margins, specimen length, distance to nearest margin, and oncologic outcomes relating to survival and recurrence rate. Recurrence was defined by evidence on imaging of local or distant disease (equivocal cases were deemed as recurrence if suggested by elevated tumor markers). Patients with metastatic disease at baseline or positive margins on pathology were excluded from this outcome since they were never truly disease-free. We also captured the following additional outcomes: time from ED to the operating room (OR), OR time, estimated blood loss (EBL), the administration of intraoperative blood products, admission and LOS in an ICU, hospital LOS, 90-day ED visit and readmission, stoma reversal rate, and time to stoma reversal. 

The groups were compared using the chi-square or Fisher’s exact tests, as appropriate, for categorical variables, and independent sample T-tests for continuous variables. Continuous variables are presented as means with standard deviations, and categorical variables as frequencies and percentages. Cumulative survival curves were plotted using the Kaplan-Meier method and statistically compared using the log-rank (Mantel-Cox) test. Statistical significance was defined as a p-value of <0.05. Statistical analysis was performed using SPSS Statistics version 29.0.2.0 (IBM Corp., Armonk, NY, USA).

## Results

The MDClone-based data registry identified 401 cases of colon resections with end ostomy based on the specified inclusion and exclusion criteria. After further data gathering, these cases were eliminated to include only resections due to CRC and underwent manual verification to ensure alignment with the study protocol. After elimination of non-CRC-related resections, non-urgent semi-elective settings, improper procedure code classification (e.g., take-back for elective colectomies, diversion ostomy surgeries), and insufficient documentation, 98 patients remained for analysis. A CRS was present in 32 (32.7%) of these cases.

Demographics and preoperative patient characteristics are outlined in Table 1. The average age was 70.1±14.6 years, and the gender distribution showed a predominance of 60 females to 38 males. The mean Charlson comorbidity score was 4.01±2.6. No significant differences were found in baseline characteristics between the groups. However, notably, a non-statistically significant trend is seen for longer ED-to-OR time in the CRS group.

**Table 1:** Patient characteristics and preoperative data GS: General surgeon, CRS: Colorectal specialist, HR: Heart rate, CRP: C-reactive protein, BE: Base excess, †Student t-test for equality of means,  ‡Pearson Chi-square

Parameters	GS n=66	CRS n=32	Test statistics value	p-value (two-sided)
Age at surgery	71.5 ± 13.8	66.9 ± 15.9	1.47 †	0.144
Gender (n, M:F)	25 : 41	19 : 13	0.07 ‡	0.794
Charlson comorbidity index	4.2 ± 2.5	3.7± 2.7	0.77 †	0.443
ED temperature (C˚)	37.1± 0.8	36.9 ± 0.6	0.83 †	0.408
ED HR (bpm)	90.9± 21.1	88.8± 20.6	0.44 †	0.662
ED mean arterial blood pressure (mmHg)	97.6 ± 16.7	102.4 ± 16.7	-1.25 †	0.215
ED WBC (count)	12.8± 7.5	12.8 ± 6.4	-0.00 †	0.999
ED CRP (mg/L)	80.7 ± 92.9	85.4 ± 122.4	-0.18 †	0.861
ED blood-gases BE (mmol/L)	-2.2 ± 4.9	-1.0 ± 4.1	-0.88 †	0.383
ED lactate (mmol/L)	2.8 ± 3.0	2.3 ± 1.2	0.56 †	0.579
Time from ED to OR (minutes)	36.4 ± 66.9	91.9 ± 329.1	-1.10 †	0.412

Intraoperative measured variables also did not differ between the groups, including OR time (3.7±1.2 vs. 4.0±1.2 hours, p=0.327), blood product administration rate (10% vs. 12.5%, p=0.725), estimated blood loss (60.1±143.5 vs. 64.5±153.1 ml, p=0.895), and intraoperative/preoperative evidence of metastatic disease (47.5% vs. 51.7%, p=0.711) for GS vs. CRS, respectively. 

Perioperative outcomes

Perioperative outcomes are outlined in Table 2. The primary outcome, which was defined as major complication rate (CD ≥ 3), was similar between the CRS and GS groups (12.5% vs. 19.7%, p=0.378), as was the overall 90-day complication rate (40.6% vs. 36.9%, p=0.724). The distribution of complication severity rated by the CD classification is outlined in Table 3, showing no differences between the groups. The 90-day mortality rate was lower in the CRS group (6.45% vs. 15.1%); however, this difference was not statistically significant (p=0.327). Additional variables relating to the index admission were also equivalent between the groups, including ICU admission rate (18.8% vs. 33.8%, p=0.123), ICU LOS (1.6±3.9 days vs. 2.5±5.3 days, p=0.433), index admission LOS (20.7±16.9 days vs. 16.8±10.6 days, p=0.160), and re-operation (3.1% vs. 3.0%, p=1.00).

**Table 2:** Intraoperative and postoperative outcomes CD: Clavien-Dindo, GS: General surgeon, CRS: Colorectal specialist, LOS: Length of stay, †Student t-test for equality of means,  ‡Pearson Chi-square, • Fisher’s Exact test, ‣ Log Rank (Mantel-cox)

Parameters	GS n=66	CRS n=32	Test statistics value	p-value (two-sided)
Intraoperative outcomes				
OR time (hours, including anesthesia)	3.7 ± 1.2	4.0 ± 1.2	0.99 †	0.327
Blood product administration during index surgery	3 (10%)	8 (12.5%)	1.00 •	1.000
Estimated blood loss (ml)	60.1 ± 143.5	64.5 ± 153.1	0.13 †	0.895
Metastatic disease in index surgery	29 (47.5%)	15 (51.7%)	0.14 ‡	0.711
Short-term postoperative outcomes				
Major complications CD≥3	13 (19.7%)	4 (12.5%)	0.78 ‡	0.378
ICU admission	22 (33.8%)	6 (18.8%)	2.38 ‡	0.123
ICU LOS (days)	2.5 ± 5.3	1.6 ± 3.9	-0.79 †	0.433
Index admission LOS (days)	16.8 ± 10.6	20.7 ± 16.9	1.42 †	0.160
90-day complications	24 (36.9%)	13 (40.6%)	0.13 ‡	0.724
Reoperation for complications	2 (3.0%)	1 (3.1%)	1.00 •	1.000
90 days postop ED visit	31 (50.0%)	9 (30.0%)	3.29 ‡	0.070
90 days postop ED readmission	18 (29.0%)	9 (28.1%)	0.01 ‡	0.927
30-day mortality rate	6 (9.1%)	2 (6.5%)	1.00 •	1.000
90-day mortality rate	10 (15.0%)	2 (6.5%)	0.33 •	0.327
Long-term postoperative outcomes				
Stoma reversal	21 (31.8%)	10 (31.3%)	0.00 ‡	0.955
Time to stoma reversal (days)	297.9 ± 124.4	304.7 ± 157.4	0.14 †	0.894
Median overall survival estimate	3.9 ± 0.9	4.8 ± 1.1	1.41 ‣	0.236

**Table 3:** CD classification complication rates CD: Clavien-Dindo, GS: General surgeon, CRS: Colorectal specialist, ‡ Pearson Chi-square, • Fisher’s Exact test

CD classification	GS n=66	CRS n=32	Test statistics value	p-value
	Frequency (%)	Frequency (%)		
0	24 (36.4%)	14 (37.5%)	0.50 ‡	0.482
1	9 (13.6%)	9 (28.1%)	0.56 •	0.556
2	20 (30.3%)	8 (25.0%)	0.30 ‡	0.586
3a	2 (3.0%)	2 (6.3%)	0.60 •	0.595
3b	2 (3.0%)	1 (3.1%)	1.00 •	1.000
4a	4 (6.1%)	0 (0.0%)	0.30 •	0.300
4b	0 (0.0%)	0 (0.0%)	-	-
5	5 (7.6%)	1 (3.1%)	0.66 •	0.660

Specimen quality outcomes

Specimen quality outcomes are summarized in Table 4. The number of lymph nodes (LN) in the specimen was significantly higher in the CRS group (24.6 ± 13.4 vs. 19.1 ± 8.8, p=0.019); however, inadequate lymphadenectomy rate (defined as fewer than 12 LN) did not differ between the groups (15.6% vs. 16.7%, p=0.991). Positive resection margins were a rare event, with all occurrences found in the GS group (four radial, one distal, 0% vs. 7.6%, p=0.11), the specimen length did not differ significantly between the groups (24.4±13.3 cm vs. 22.3±11.5 cm, p=0.436) nor did the distance to the nearest proximal/distal margin (4.5±2.9 cm vs. 4.8±3.1 cm, p=0.752). Also, when combining inadequate lymphadenectomy and positive margins into a single outcome as a “suboptimal resection,” there was no significant difference between the groups (16.1% vs. 25.0%, p=0.329).

**Table 4:** Specimen quality metrics and oncological outcomes LN: Lymph node, LNY: Lymph node yield, † Student t-test for equality of means,  ‡ Pearson chi-square, • Fisher’s exact test

Parameters	GS n=66	CRS n=32	Test statistics value	p-value (two-sided)
Inadequate LN excision (less than 12)	11 (16.7%)	5 (15.6%)	0.02 ‡	0.991
Number of LNs excised	19.1 ± 8.9	24.6 ± 13.4	2.38 †	0.019
Positive margins for cancer surgery	5 (7.6%)	0 (0.0%)	0.11 •	0.110
Distance to nearest proximal/distal margin (cm)	4.8 ± 3.1	4.5 ± 2.9	-0.32 †	0.752
Suboptimal resection (either LNY<12 or positive margins)	16 (25.0%)	5 (16.1%)	0.95 ‡	0.329
Specimen length (cm)	22.3 ± 11.5	24.4 ± 13.3	0.78 †	0.436
Recurrence rate	6 (22.2%)	1 (6.7%)	0.39 •	0.390
Median overall survival estimate	3.9 ± 0.9	4.8 ± 1.1	1.41 ‣	0.236

Oncologic and long-term outcomes

**Figure 1:**
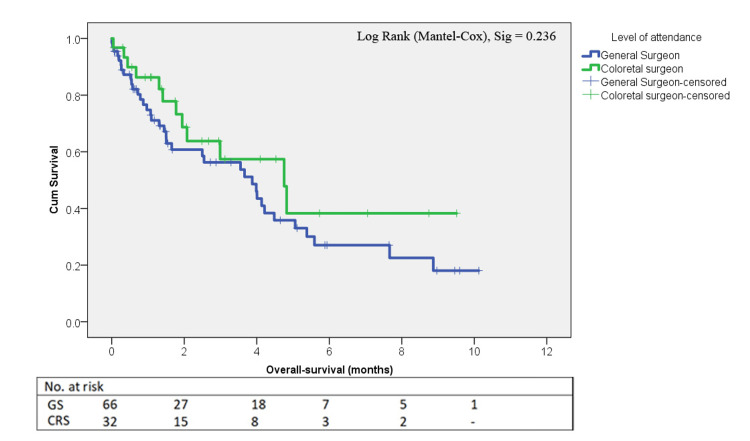
Kaplan-Meier survival curve according to level of specialty GS: General surgeon, CRS: Colorectal specialist

Median follow-up was 1.5±2.67 years, during which there was not a meaningful difference in overall survival (log-rank p=0.236). Figure 1 shows the Kaplan-Meier survival curves. Although not statistically significant, the CRS group survival curve was consistently higher than that of the GS group. Recurrence rates were also similar (16% vs. 25%, p=0.390), showing a trend favoring CRS. Ostomy closure rates (31.3% vs. 31.8%, p=0.955) and time to closure (304.7 ± 157.4 vs. 297.9 ± 124.4 days, p=0.894) were similar between the groups. 

## Discussion

In this study, we did not find a difference in perioperative complications following emergent colon resection with end stoma when comparing surgeries performed by CRS surgeons to those performed by GS. There were some potentially meaningful, though non-significant, trends in the perioperative outcomes favoring the CRS group. These included lower perioperative mortality at both 30 and 90 days following surgery, a lower rate of severe complications, and a lower rate of ED visits following the index admission. It is possible that with a larger cohort, some of these findings would have become statistically significant.

There was a difference in the quality of resected specimens between the two groups. The LNY was meaningfully higher in the CRS group, and all incidences of positive resected margins occurred in the GS group, although the latter finding was not statistically significant. There was an additional potentially meaningful, though not statistically significant, trend of a lower rate of inadequate resection, defined as either positive margins or LNY < 12 or both, in the CRS group.

We did not find a statistically meaningful difference in oncological outcomes between the groups. However, trends once again favored the CRS group both for median (mean) overall survival and rate of recurrence. In addition, the Kaplan-Meier curves show that the survival of the CRS group is consistently higher than the GS group.

The findings of this study suggest that urgent colectomies for colon cancer, when performed by a specialized CRS surgeon or GS, result in comparable short-term perioperative outcomes. Both groups exhibited similar rates of overall complications, major complications (CD ≥ 3), and mortality. The study also highlighted some practical aspects of surgical performance, including estimated blood loss, duration of surgery, and ICU-related metrics, all of which were similar between the two groups. These findings reinforce the notion that GS are well-equipped to perform urgent CRC-related colectomies from a technical standpoint and provide surgical care that is largely equivalent to CRS without compromising patient safety or other outcomes.

However, certain differences emerged, particularly regarding specimen quality metrics, that warrant further exploration. The most significant finding in this study was a higher LNY in the CRS group compared to the GS group. A higher LNY has been linked to more accurate staging and potentially improved long-term outcomes in CRC surgery, particularly in terms of disease-free and overall survival [19]. The CRS group harvested an average of 24.6 lymph nodes, significantly exceeding the yield from the GS group (19.1, p=0.019), although the rate of inadequate lymphadenectomy (fewer than 12 LNs) was similar between the two groups. This could suggest that, while GS can achieve adequate resections, CRS surgeons may offer a more thorough lymphadenectomy, potentially influencing long-term outcomes. Moreover, the presence of positive surgical margins, all observed in the GS group, could have implications for local recurrence rates and survival, although no significant differences were observed in overall survival within the follow-up period of this study. These oncological quality metrics highlight the importance of CRS involvement, especially in more complex or advanced cases of CRC, which is in keeping with previously discussed studies [6,7].

Despite the comparable perioperative complication rates between the two groups, non-statistically significant trends toward better outcomes in the CRS group were exhibited in several aspects. These findings should be interpreted cautiously and warrant further investigation. We found a lower 90-day mortality in the CRS group (6.45% vs. 15.1%, p=0.225) as well as a lower absolute major complications rate, a lower ICU admission rate, and absolute lower rates of repeated ED visits, all suggesting the possibility of superior short-term outcomes with CRS involvement. Additionally, the CRS group also consistently showed a higher overall survival curve according to the Kaplan-Meier survival analysis, not reaching statistical significance (log-rank p=0.236). Although these differences did not reach statistical significance, likely due to the small sample size, they raise important questions about the potential benefits of specialized care in emergency settings on both short- and long-term outcomes. These findings are consistent with previous literature, which has demonstrated that surgeon specialization can improve outcomes in elective colorectal surgeries [20]. However, the fact that these trends did not achieve statistical significance underscores the need for larger studies with adequate power to re-evaluate the observed differences, as they may be clinically meaningful.

Several limitations of this study must be acknowledged. The study is subject to biases inherent to the retrospective design, including selection and recall biases. Selection bias may have played an additional role, as more complex cases may have been directed to CRS, skewing the outcomes. The relatively small sample size may have been underpowered to detect meaningful differences in some outcomes, particularly concerning survival and complication rates. Furthermore, the single-center nature of the study limits the generalizability of the findings. Future studies should address these limitations by incorporating a larger sample size, a prospective design, and multicenter data collection to confirm or refute the trends observed here.

## Conclusions

While urgent colectomies performed by GS yield outcomes comparable to those performed by CRS surgeons in many respects, the CRS presence is associated with a non-significant trend towards improved outcomes. The involvement of a CRS is also associated with differences in specimen quality metrics, notably a higher LNY and the absence of positive surgical margins. Although these findings did not translate into statistically significant differences in survival within the study period, they raise important questions about the impact of specialization on long-term patient prognosis. The study’s limitations, as elaborated above, underscore the need for larger, prospective, multicenter trials to validate these observations. Future research should aim to clarify whether the trends identified in this study represent true clinical advantages of CRS involvement in emergent CRC surgeries.
